# Composition-Dependent Structural, Phonon, and Thermodynamical Characteristics of Zinc-Blende BeZnO

**DOI:** 10.3390/ma18133101

**Published:** 2025-07-01

**Authors:** Devki N. Talwar, Piotr Becla

**Affiliations:** 1 Department of Physics, University of North Florida, 1 UNF Drive, Jacksonville, FL 32224, USA; 2 Department of Physics, Indiana University of Pennsylvania, 975 Oakland Avenue, 56 Weyandt Hall, Indiana, PA 15705, USA; 3 Department of Materials Science and Engineering, Massachusetts Institute of Technology, Cambridge, MA 02139, USA; becla@mit.edu

**Keywords:** novel ZnO, BeO, ZnBeO, Raman scattering, Fourier transform infrared spectroscopy, rigid-ion-model, phonon dispersions, thermodynamical properties

## Abstract

Both ZnO and BeO semiconductors crystallize in the hexagonal wurtzite (wz), cubic rock salt (rs), and zinc-blende (zb) phases, depending upon their growth conditions. Low-dimensional heterostructures ZnO/Be_x_Zn_1-x_O and Be_x_Zn_1-x_O ternary alloy-based devices have recently gained substantial interest to design/improve the operations of highly efficient and flexible nano- and micro-electronics. Attempts are being made to engineer different electronic devices to cover light emission over a wide range of wavelengths to meet the growing industrial needs in photonics, energy harvesting, and biomedical applications. For zb materials, both experimental and theoretical studies of lattice dynamics $\omega_{j}\left(\vec{q}\right)$ have played crucial roles for understanding their optical and electronic properties. Except for zb ZnO, inelastic neutron scattering measurement of $\omega_{j}\left(\vec{q}\right)$ for BeO is still lacking. For the Be_x_Zn_1-x_O ternary alloys, no experimental and/or theoretical studies exist for comprehending their structural, vibrational, and thermodynamical traits (e.g., Debye temperature $\Theta_{D}\left(T\right);$ specific heat $C_{v}\left(T\right))$. By adopting a realistic rigid-ion model, we have meticulously simulated the results of lattice dynamics, and thermodynamic properties for both the binary zb ZnO, BeO and ternary Be_x_Zn_1-x_O alloys. The theoretical results are compared/contrasted against the limited experimental data and/or ab initio calculations. We strongly feel that the phonon/thermodynamic features reported here will encourage spectroscopists to perform similar measurements and check our theoretical conjectures.

## 1. Introduction

Highly efficient and flexible devices are essential for the technological needs of micro- and nano-electronics in modern society. Researchers working in this area have devoted their efforts to design electronic device structures by acquiring cheap, superior-quality, novel, and non-toxic materials to meet the escalating demands in the commercial market. The revolution in photonics that took place over the past few decades has exploited different types of wide bandgap (WBG) semiconductor materials [1,2,3,4,5,6,7,8,9,10,11,12,13,14,15,16]. Progress made in the opto-electronics has changed our life in a way that has never been envisaged since the ingenuity of silicon-based transistors. Efforts to acquire partially covalent III-nitrides (or III-Ns: BN, AlN, GaN, InN) and partially ionic II-oxides (or II-Os: BeO, MgO, ZnO, CdO) began in the late 1990s [10,11,12,13,14,15,16]. Earlier difficulties for growing III-Ns were linked either to their low dissociation temperatures T, and/or the lack of suitable substrates. Despite these issues, Nakamura (1993) successfully developed the highly efficient and bright blue-ultraviolet (UV) light-emitting diode (LED) using InGaN material as an emitter [16]. A few years later, the progress made in the improvements of new epitaxial techniques have demonstrated achieving ZnO-based laser diodes (LDs) and LEDs [16].

Advancement in the growth methods for preparing II-Os have helped realizing high-quality single crystals, large area ultrathin films, and various nanostructures. Unlike III-Ns, the oxide materials are grown relatively easily by combining elements from the group II^B^ alkaline-earth metals (e.g., beryllium Be, magnesium Mg, cadmium Cd, zinc Zn) with a nontoxic oxygen O of group VI^A^ [16]. In recent years, oxide materials have gained significant attention due to their superior basic properties including high optical transparency, WBG, and electrical conductivity for attaining the next generation photonics. Many physical and chemical properties of III-Ns have revealed similarities with the members of II-Os (viz., BN→ BeO; AlN, (GaN, InN) → MgO, (ZnO, CdO)) [17]. Room temperature (RT) bandgap $E_{g}$ ~ 3.39 eV of GaN and SiC is found comparable to that of ZnO ~ 3.37 eV [18,19,20,21,22]. Very close lattice constants of the two materials (GaN, ZnO) are used as appropriate substrates [16] for epitaxial growth of GaN on ZnO (or ZnO on GaN). Electronic devices based on III-Ns and II-Os have covered nearly the same wavelength range. However, ZnO is projected superior to GaN to fabricate lower threshold LDs, with excitons operating at higher efficiencies. The exciton binding energy of ZnO ~ 60 meV is nearly twice than that of GaN (~26 meV) [16]. This has made ZnO promising to engineer high-efficiency and low-cost UV photodetectors. So far, the highly textured ZnO films have demonstrated optically pumped UV lasing at RT. Before ZnO offered a significant contribution on the world of micro- and nano-electronics, it is important to achieve a reproducible p-type epilayers. Despite a substantial improvement in the growth processes, consistent replication of p-type ZnO films has not yet been attained. In intentionally doped ZnO, many intrinsic defects, e.g., Zn interstitial $\left({Zn}_{i}\right)$ and O vacancy $\left(V_{O}\right)$ are ascribed for compensating p-type dopants. On the contrary, ${Zn}_{i}$ and $V_{O}$ exhibit low formation enthalpies [16]. Therefore, it is necessary to resolve this bottleneck issue to achieve high-quality ZnO epilayers with low n-type background charge carriers. Obviously, one needs to identify the site selectivity of both intrinsic impurities and intentionally p-doped defects either experimentally by using optical spectroscopy or theoretically by exploiting lattice dynamical methods [18].

Besides ZnO, the BeO material is another distinctive member of the II-Os family. It exhibits high thermal conductivity, exceptional hardness, large bond strength, a high melting point of ~2570 °C, radiation resistance, and higher dielectric constant, despite having a large bandgap $E_{g}$ ~ 10.6 eV [16]. High thermal conductivity of BeO makes it one of the best heat-conducting compounds among ceramics [23]. The use of BeO in devices can resist high voltage and high temperature without breakdown. It has been effectively employed as an electronic substrate and insulator for rapid heat dissipation to maintain performance and longevity of different electronic components. Since BeO is chemically inert to most acids and alkalis, it offers excellent corrosion resistance to acids, solvents, and even molten metals. For achieving high-efficiency LEDs, one requires high-quality and stable barrier layers of a material with $E_{g}$ larger than ZnO. In micro- and nanoelectronics, the use of low-dimensional heterostructures (LDHs) [viz., BeO/ZnO, ZnO/BeZnO multi quantum wells (MQWs), superlattices (SLs)], and ${Be}_{x}{Zn}_{1-x}O$ ternary alloys are considered quite promising [24,25]. Precise tuning of $E_{g}$ by alloy composition x makes ${Be}_{x}{Zn}_{1-x}O$ epifilms important to fabricate UV photodetectors with different cut-off edges. Due to the large lattice mismatch between BeO and ZnO, the preparation of ${Be}_{x}{Zn}_{1-x}O$ epifilms for all x has been a challenge [24,25]. Limited ZnO/BeZnO-based devices are available to manage the temperature variation for ensuring operational stability and efficiency. Considering the emerging interests of ${Be}_{x}{Zn}_{1-x}O$ ternary alloys in LDHs, it seems quite likely that better quality epilayers with higher composition x will be produced soon.

In recent years, the preparation of II-Os epifilms has been successfully achieved using various epitaxial and chemical techniques [26,27,28,29,30,31,32,33,34,35,36,37]. The methods that are frequently employed for their growth included MOCVD [26,27,28,29,30], PA-MBE [31,32,33,34,35], thermal decomposition, sol–gel and co-precipitation, among others [27,28,29,36,37]. Different procedures have suggested considerable flexibility in making ultrathin films by controlling their size, dimension and morphology [24,38,39,40,41,42,43,44,45,46]. Syntheses and characterization of ternary A_x_B_1-x_O and quaternary A_x_B_1-x-y_C_y_O (with A, B, C ≡ Be, Mg, Zn, Cd) alloys [47,48,49,50,51,52,53,54] have contributed to designing various device structures [55,56,57,58]. The incorporation of ultrathin films of binary and/or ternary alloys in MQWs and SLs has offered endless possibilities to design/fabricate opto-electronic devices. ZnO/A_x_B_1-x_O are commonly used to engineer high-power heterojunction field effect transistors (HFET); high-electron mobility transistors (HEMT); biosensors; UV detectors; and single-chip devices [16]. In LDHs, different methods are frequently employed to assess their surface morphology, structural/crystalline quality [24,38,39,40,41,42,43,44,45,46], and electronic and vibrational behavior. The measurements that are commonly used to study various functionalities of ZnO/A_x_B_1-x_O are, reflection high-energy electron diffraction (RHEED); scanning electron microscopy (SEM) [24,38,39]; photoluminescence (PL); transmission electron microscopy (TEM); cathodoluminescence (CL) [40,41]; spectroscopic ellipsometry (SE); Hall or van der Pauw measurements [43,44,45]; Raman scattering spectroscopy (RSS) [46,47,48,49,50,51,52,53,54]; Fourier transform infrared (FTIR) and/or transmission spectroscopy [48,49]; Rutherford backscattering (RBS); and atomic force microscopy (AFM), among others [42,43,44,45].

Despite the successful growth [26,27,28,29,30,31,32,33,34,35,36,37] and characterization [24,38,39,40,41,42,43,44,45,46,47,48,49,50,51,52,53,54,55,56,57,58], there are still many basic issues of II-O-based device structures that remain unresolved [16]. The structural, electronic, elastic, and vibrational traits [24,38,39,40,41,42,43,44,45,46] of epitaxially grown BeO, ZnO, and ${Be}_{x}{Zn}_{1-x}O$ epilayers have not yet been thoroughly investigated. To obtain energy–momentum relationships of phonons in solids, an inelastic neutron scattering (INS) spectroscopy has been the most powerful and frequently used technique [54]. Careful analysis of energy lost and/or gained by scattered neutrons is employed for determining the phonon dispersions $\omega_{j}\left(\vec{q}\right)$ of zb ZnO [54]. In the absence of INS studies, non-destructive Raman and/or FTIR spectroscopies [48,49,50,51,52,53,54,55,56,57,58] have been exploited for assessing phonon frequencies at high critical points in the BZ. In crystals with free charge carriers, RSS has also been successfully used for understanding carrier–phonon interactions and phonon-assisted optical transitions [54,55,56,57,58]. Except for a zinc-blende (zb) ZnO and a wurtzite (wz) ZnO, no INS and Raman studies exist of $\omega_{j}\left(\vec{q}\right)$ for the zb BeO material. The vibrational features of ZnO have provided valuable information for their structural, electronic, and crystalline qualities [54,55,56,57,58]. In LDHs, the RSS can be extremely valuable in probing the vibrational data on alloying, strain, and intermixing of different layers. Such features in MQW- and SL-based II-Os are crucial to understanding their roles in different device performance. 

Due to limited experimental data on the binary/ternary alloys of novel II-Os, many researchers have developed theoretical methods to effectively synthesize/predict their structural, electronic, vibrational, and thermodynamic traits [59,60,61,62,63,64,65,66,67]. By exploiting a commercial package of ABINIT software package and/or Quantum-Espresso program, the theoretical results are available on the electronic properties of binary wz ZnO and BeO materials [59,60,61,62]. These simulations have employed the first-principles plane-wave self-consistent pseudopotential methods [60,61,62] in the local-density approximation (LDA) [63,64,65,66,67]. Efforts are made to acquire trends in correlating the microhardness of II-Os with their bulk, shear, and Young’s moduli [17]. Comprehending the lattice dynamics of ZnO, BeO, and ${Be}_{x}{Zn}_{1-x}O$ is of fundamental interest. Phonon characteristics [16] are frequently considered for evaluating the efficiency of different device structures in microelectronics, optoelectronics, heat transport, and quantum electronics. Very few calculations exist on the lattice dynamics of zb ZnO and BeO [60,61,62]. Some published reports on the electronic and vibrational characteristics available in the literature are either inconsistent and/or questionable [61,62]. For instance, Chibueze [61] and Zafar et al. [62] have adopted an ab initio approach to study the mechanical, structural, phonon, and electronic properties of zb ZnO material in the framework of a density function theory (DFT) by using a generalized gradient approximation (GGA). The author [61] has considered a revised Perdew–Burke–Ernzerhof GGA method and claimed achieving degenerate phonon energies at the center of the BZ (i.e., $\omega_{LO(\Gamma )}$ = $\omega_{TO(\Gamma )}$ ∼379 cm^−1^ near $\vec{q}$ → 0). For zb ZnO, this outcome is in complete disagreement with the existing INS [54] and Raman scattering spectroscopy results [48,49,50,51,52,53,54,55,56,57] of phonon dispersions $\omega_{j}\left(\vec{q}\right)$ reported by Serrano et al. [54] and LDA simulations [66]. Evaluating the importance of thin zb ZnO, BeO, and ${Be}_{x}{Zn}_{1-x}O$ epifilms in LDHs-based ZnO/Be_x_Zn_1-x_O structures for thermal management devices, realistic calculations of lattice dynamics $\omega_{j}\left(\vec{q}\right),$ density of states (DOS) $g\left(\omega \right)$, Debye temperature $\Theta_{D}$(T), specific heat $C_{v}\left(T\right)$, Grüneisen constants $\gamma \left(T\right)$, and linear thermal expansion coefficient $\alpha \left(T\right)$ are necessary [68,69,70,71].

This paper aims to report on the results of a methodical study for understanding the structural, phonon, and thermodynamical properties of binary zb ZnO, BeO. and ternary ${Be}_{x}{Zn}_{1-x}O$ alloys. In Section 2.1 and Section 2.2, different crystal structures and epitaxial growth of II-Os are described. For designing XO-based device structures, the zb phase played an important role due to its unique optical and electronic properties [16]. Specifically, the zb structure offers potential advantages like lower carrier scattering and higher doping efficiencies as compared to the commonly available wz structure. These features have made the zb ZnO material suitable for applications in optoelectronics and nanoelectronics. Some ab initio calculations provided conflicting results [61,62] of structural, elastic, electronic, and lattice dynamical $\omega_{j}\left(\vec{q}\right)$ properties for the zb and wz phases of ZnO and BeO. Absolutely no efforts have been made to comprehend the vibrational and thermodynamic traits of ${Be}_{x}{Zn}_{1-x}O$. Among many nonmetallic solids, the phonons in BeO are suggested to be the main heat carriers [16]. Accurate description of lattice dynamics in ternary alloys is vital, as it plays important roles for determining phase transitions, electron–phonon interactions, and transport coefficients. The vibrational characteristics of perfect binary zb ZnO and BeO materials are reported here by adopting a realistic rigid-ion model (RIM) [68] (cf. Section 3, Section 3.1.1, Section 3.1.2 and Section 3.1.3). A generalized Greens function (GF) theory [69] is considered in the virtual crystal approximation (VCA) for studying the phonons in mixed Be_x_Zn_1-x_O ternary alloys. The RIM method used here is succinctly outlined in Section 3.1.1, Section 3.1.2 and Section 3.1.3. The model includes both the short-range and long-range Coulomb interactions. For estimating the interatomic force constants (IFCs), we have used (cf. Section 3.1.1) values of critical-point phonon frequencies of zb ZnO and BeO materials at a few critical points (Γ, X and L) as input, while their elastic $c_{ij}$ constants and equilibrium lattice-constants $a_{0}$ are employed as constraints [70,71]. The calculated values of IFCs are systematically exploited in the RIM framework to comprehend the lattice dynamics, structural, and thermodynamic characteristics of ${Be}_{x}{Zn}_{1-x}O$ ternary alloys. The results of RIM calculations are compared (see Section 4) with the experimental and/or ab initio studies [72,73,74,75,76,77,78,79,80]. In highly ionic zb BeO crystal, we have noticed a large splitting between $\omega_{LO(\Gamma )-}$ $\omega_{TO(\Gamma )}$ modes at the center of BZ. Unlike zb ZnO, the calculated phonon dispersions of zb BeO exhibited atypical behavior like that in the cubic BN and diamond [81,82,83]—revealing the longitudinal acoustic (LA) modes falling between the LO-TO phonons. A possible explanation for this abnormal trend is linked to the combined effects of short bond length and strong electron localization due to the small core size of the electronic charge near the Be atom in BeO. The concluding remarks are presented in Section 5. We strongly feel that the phonon and thermodynamic features reported here will encourage spectroscopists to perform similar measurements and check our theoretical conjectures.

## 2. Material Properties of II-Os

### 2.1. Crystal Structures of ZnO and BeO

**Figure 1:**
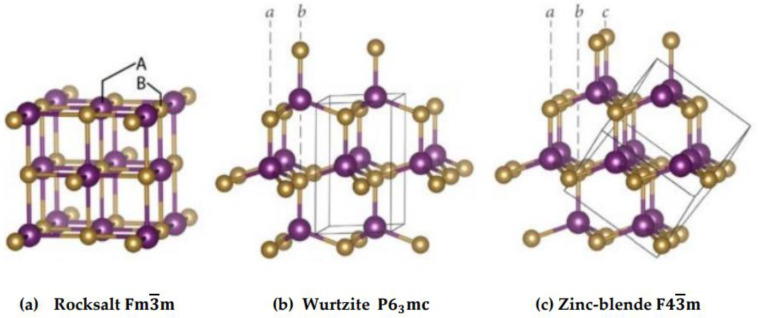
Three different crystal structures of the novel XO (X = Zn, Be) materials: (**a**) the cubic rock salt ($Fm\bar{3}m$), (**b**) wurtzite ($P6_{3}mc$), and (**c**) zinc-blende ($F4\bar{3}m)$ in the Strukturbericht designation. The small-size gold-colored balls represent the O atoms, while the large-size purple-colored balls signify the X atoms. The letters A and B in Figure (**a**) signify the X and O atoms. The symbols *a* and *b* in Figure (**b**) and *a*, *b*, and *c* in Figure (**c**) show the different stacking sequences of the close-packed planes of O (gold) and X (purple) atoms in the wurtzite and zinc-blende structures, respectively.

The XO (X ≡ Be, Zn) materials are known to have a non-centrosymmetric wurtzite (wz) crystal structure (see Figure 1b) of the B_4_ phase or the ${P6}_{3}mc{(C}_{6v}^{4})$ space group with polar surfaces at ambient (i.e., temperature T and pressure P) conditions. The wz structure is considered having two interpenetrating hexagonal close packed (hcp) sublattices of cation (X) and anion (O) displaced by the length of cation-anion bond in the [0001]-direction. Ultrathin metastable XO films of the cubic zinc-blende (zb) phase with a space group $F\bar{4}3m\;{(T}_{d}^{2})$ is possible (see Figure 1c) if prepared on the GaAs (001), (111), and/or Si substrates [16]. The zb materials present a mixture of the tetrahedral covalent and ionic bonding. At high pressure, the XO materials transform to the NaCl-like/rocksalt (rs) or B_1_ polymorph of $Fm3m{(O}_{h}^{5})$ space group (see Figure 1a).

In principle, the XO materials are generally classified as highly mismatched due to a large difference in lattice constants and electronegativity between the X cation (i.e., low electronegative metallic atom) and the O anion (i.e., high electronegative atom). Such disparity can cause energetically favorable lattice defects such as cation/anion vacancies [18]. These intrinsic defects are suggested for trigerring localized energy states within the WBG II-Os. Such impurity levels are known to be strongly influencing the electronic and optical properties [18]. The formation of preferential intrinsic defects such as $V_{O}$ is predominantly dependent on the position of the Fermi level with respect to the charge neutrality level, i.e., the Fermi stabalization energy [16].

### 2.2. Epitaxially Grown II-O Films

The epitaxially grown XO films on most substrates yield a stable wz structure. There are reports that also suggest achieving epifilms with the zb and rs phases [16]. Investigation of high-pressure phase transitions in XO [24,38,39,40,41,42,43,44,45,46,47,48,49,50,51,52,53,54,55,56,57,58] has been the subject of intense experimental and theoretical efforts [59,60,61,62,63,64,65,66,67,68,69,70,71]. These studies have revealed appropriate sequences of pressure-dependent transformations from wz → zb → rs structures. Depending on the approximations used in first-principle calculations [61,62], significant divergences exist in the predictions of their basic characteristics. Efforts have been made earlier for studying the elastic, thermodynamic, lattice dynamics, and pressure-dependent properties [72,73] in different semiconductors. Very few attempts have been made; however, to explore the lattice dynamics of binary XO and alloy epifilms. 

## 3. Vibrational Properties

### 3.1. Lattice Dynamics

Two types of theoretical approaches are frequently considered for studying the lattice dynamics of zb semiconductors. The techniques include (a) a microscopic or first-principle method [59,60,61,62,63,64,65,66,67], which employs an ionic potential screened by electron gas to study the structural/vibrational and elastic properties, and (b) a macroscopic technique, which uses phenomenological models [68,69,70,71] in terms of the general IFCs. For systematically studying the phonon dispersions $\omega_{j}(\vec{q})$ and thermodynamic properties at ambient conditions of zb ZnO and BeO materials, we have adopted a realistic RIM [68] (cf. Section 3.1.2). The optimized set of appropriate IFCs are carefully obtained in Section 3.1.1.

#### 3.1.1. Interatomic Force Constants

Unique optimization procedures described elsewhere [71] are applied here for estimating the RIM interatomic force constants for the binary zb ZnO and BeO materials. In calculating IFCs, we have used accurate values of experimental [54] and/or theoretical [60] phonon frequencies at high critical points (Γ, X and L) as input (see Table 1), while the elastic constants $c_{ij}$ and equilibrium lattice-constants $a_{0}$ are employed as constraints.

In column 3 of Table 1, we have listed the phonon frequencies as well as the lattice and elastic constants of zb ZnO [54]. A similar ab initio data [60] for the zb BeO is reported in column 5. Columns 2 and 4 register our RIM-calculated phonon frequencies. The lattice constant and elastic constants are obtained after achieving the optimized IFCs.

#### 3.1.2. Rigid-Ion Model

The vibrational properties of XO materials have played valuable roles for assessing important basic characteristics including the phase transitions, transport coefficients, electron–phonon interactions, and polaron behavior [16]. In Be_x_Zn_1-x_O ternary alloys, the knowledge of phonons and the thermodynamic, optical, and structural traits are crucial for correlating them to their different microscopic attributes. A realistic RIM [68] is adopted here for simulating phonon dispersions $\omega_{j}(\vec{q})$, one-phonon DOS $g\left(\omega \right),$ and the thermodynamic properties of binary zb ZnO, BeO and ternary Be_x_Zn_1-x_O alloys.

The RIM that we have considered here includes both the short-range (up to second nearest neighbors) and long-range Coulomb interactions [68]. An atom in a zb crystal is identified by two indices, namely *l* and *κ*. The term *l* represents the number of unit cells while *κ* signifies the two types of atoms (i.e., *κ* = 1 (O) and *κ* = 2 (X) in XO materials). Following Kunc [68], the polarization in crystals is determined by the displacements of point ions from their equilibrium positions anticipating them to be rigid and non-polarized. The quantities of interest in RIM are the force constants ${\overset{⃡}{\Phi }}^{sC}\left[\equiv {\overset{⃡}{\Phi }}^{s}+{\overset{⃡}{\Phi }}^{C}\right]$ or dynamical ${\overset{⃡}{D}}^{sC}\left[\equiv {\overset{⃡}{D}}^{s}+{\overset{⃡}{D}}^{C}\right]$ matrices [68]. Ten short-range (${\overset{⃡}{\Phi }}^{s}$) force constants and one long-range Coulomb interaction (${\overset{⃡}{\Phi }}^{C})\;Z_{eff}(\equiv Z_{Κ}e)$ are involved. In the harmonic approximation, the lattice vibrations $\omega_{j}(\vec{q})$ are obtained by solving the following equations of motion [68]:

(1) $$\omega_{j}^{2}(\vec{q})e_{\alpha }(\kappa |\vec{q}j)=\sum_{\kappa \prime \beta }D_{\alpha \beta }^{sC}(\kappa \kappa \prime |\vec{q})e_{\beta }(\kappa \prime |\vec{q}j);\kappa ,\kappa \prime =1,2,$$

where $D_{\alpha \beta }^{sC}(\kappa \kappa \prime |\vec{q})\left[\equiv D_{\alpha \beta }^{s}(\kappa \kappa \prime |\vec{q})+D_{\alpha \beta }^{C}(\kappa \kappa \prime |\vec{q})\right]$ represents the dynamical matrix comprising the short-range $D_{\alpha \beta }^{s}(\kappa \kappa \prime |\vec{q})$ and long-range Coulomb $D_{\alpha \beta }^{C}(\kappa \kappa \prime |\vec{q})$ interactions. For each mode frequency $\omega_{j}\left(\vec{q}\right),$ the components of eigenvectors $e_{\alpha }(|\vec{q}j)$ in Equation (1) satisfy the familiar orthogonality [68]

(2a) $$\sum_{\alpha \kappa }e_{\alpha }^{*}(\kappa |\vec{q}j)e_{\alpha }(\kappa |{\vec{q}j}^{\prime })=\delta_{jj^{\prime }}\;,$$

and closure relations

(2b) $$\sum_{j}e_{\alpha }^{*}(\kappa \prime |\vec{q}j)e_{\beta }(\kappa |{\vec{q}j}^{\prime })=\delta_{\prime }\delta_{\alpha \beta }\;.$$

Once the optimized set of IFCs $[A,B,C_{\kappa },D_{\kappa },E_{\kappa },F_{\kappa }$, and $Z_{eff}(\equiv Z_{\kappa }$e)] are evaluated (see Table 2) for the binary zb ZnO and BeO (cf. Section 3.1.2) materials, it is straightforward to simulate $\omega_{j}(\vec{q})$ and density of states (DOS) $g\left(\omega \right)$ using Equation (1).

#### 3.1.3. Phonon-Induced Thermal Characteristics

In zb II-Os, the study of $\omega_{j}(\vec{q})$ plays a prominent role for comprehending several important phenomena, including thermal expansion α(T), Grüneisen parameters γ(T), heat capacity $C_{v}\left(T\right)$, entropy, Debye temperature $\Theta_{D}\left(T\right),$ etc. [16]. For semiconductor materials, the phonons are also linked to many of their physical characteristics such as elastic and mechanical properties. Accurate knowledge of thermodynamical traits for II-Os is important not only from a basic standpoint but also to design and engineer devices for applications in advanced semiconductor technology.

By using RIM in conjunction with a quasi-harmonic approximation (QHA), one can perform systematic simulations to obtain structural, vibrational, and thermodynamic properties [70,71]. Phonon contributions to the Helmholtz free energy, entropy, and constant-volume-specific heat are determined (cf. Section 3.1.4). The RIM calculations (cf. Section 4) of $\omega_{j}(\vec{q})$, $C_{v}\left(T\right),$ and $\Theta_{D}\left(T\right)$ for zb ZnO, BeO, and Be_x_Zn_1-x_O alloys have agreed reasonably well with the existing experimental/theoretical data [72,73,74,75,76,77,78,79,80].

#### 3.1.4. Specific Heat and Debye Temperature

For II-O semiconductors, the complete knowledge of phonon dispersions $\omega_{j}\left(\vec{q}\right)$ and density of states $g\left(\omega \right)$ using RIM makes it possible to calculate different thermodynamical properties as a function of temperature T. For $C_{v}\left(T\right),$ it is necessary to evaluate the Helmholtz free energy $\Phi_{vib}$ of the atomic vibrations $\omega_{j}\left(\vec{q}\right)$ [73]:

(3) $$\Phi_{vib}=\frac{1}{2}\sum_{j\vec{q}}\hbar \omega_{j}\left(\vec{q}\right)+k_{B}T\sum_{j\vec{q}}ln\left[1-e^{-\frac{{\hbar \omega }_{j}\left(\vec{q}\right)}{k_{B}T}}\right],$$

where $k_{B}$ is the Boltzmann constant. The entropy ($S_{vib}$) of vibrating lattice is linked to the Helmholtz free energy via $S_{vib}\equiv {-\frac{\partial \Phi_{vib}}{\partial T}|}_{V}$. Clearly, the knowledge of $\Phi_{vib}$ and $S_{vib}$ allows the calculations of internal energy U of the crystalline lattice by using $\Phi_{vib}=U-S_{vib}T.$ The heat capacity at a constant volume $C_{v}\left(T\right)$ can be obtained either by exploiting $\left(\equiv {T\left(\frac{\partial S_{vib}}{\partial T}\right)|}_{V}\right)$ or $\left(\equiv {-T\left(\frac{\partial^{2}\Phi_{vib}}{\partial T^{2}}\right)|}_{V}\right)$. In the QHA, and using Equation (3), $C_{v}\left(T\right)$ is obtained as a function of T [73] i.e.,

(4) $$C_{v}\left(T\right)=\sum_{j\vec{q}}k_{B}{\left(\frac{{\hbar \omega }_{j}\left(\vec{q}\right)}{k_{B}T}\right)}^{2}\left(\frac{e^{\frac{{\hbar \omega }_{j}\left(\vec{q}\right)}{k_{B}T}}}{{\left[e^{\frac{{\hbar \omega }_{j}\left(\vec{q}\right)}{k_{B}T}}-1\right]}^{2}}\right).$$

From the Debye’s equation

(5) CvT=9rkBTΘD(T)3∫0ΘD(T)Tx4exex−12dx,

one can simulate $\Theta_{D}(T)$ for the zb XO materials. In Equation (5), the term r signifies the number of atoms per unit cell.

### 3.2. Lattice Dynamics of Ternary A_x_B_1-x_O Alloys

Composition-dependent phonon dispersions $\omega_{j}(\vec{q})$ for the mixed zb A_x_B_1-x_O crystals are simulated as a function of x (0 ≤ *x* ≤ 1) (cf. Section 4) by adopting a generalized GF theory in the VCA [69]. For a one-dimensional ternary A_x_B_1-x_O alloy with two interpenetrating sublattices, Kutty has developed a GF approach and derived the following dynamical matrix equation [69]:

(6) $$\left|\left(\omega^{2}M_{\alpha }^{\mu }-\Phi_{\alpha \alpha }^{\mu \mu }\right)\delta_{\alpha \beta }\delta_{\mu \nu }-C_{\alpha }^{\mu }\Phi_{\alpha \beta }^{\mu \nu }\left(\vec{q}\right)\right|=0,$$

by postulating that sublattice 1 occupies O atoms while sublattice 2 is randomly acquired by atoms of type A and B having concentrations (x) and 1 − x, respectively. In Equation (3), $C_{1}^{O}=1$, $C_{2}^{B}=(1-x)$, $C_{2}^{A}=x$, $M_{1}^{O}=m_{O}$, $M_{2}^{B}=m_{B}$, ${andM}_{2}^{A}=m_{A},$ and the term $\Phi_{\alpha \beta }^{\mu \nu }$ represents the IFCs. By substituting these parameters in Equation (6), it gives [69]

(7) $$\left|\begin{array}{ccc} \omega^{2}m_{C}-\Phi_{11}^{11} & -\Phi_{12}^{12}(\vec{q}) & -\Phi_{12}^{12}(\vec{q})\\ -{(1-x)\Phi }_{21}^{21}(\vec{q}) & \omega^{2}m_{Y}-\Phi_{22}^{22} & -{(1-x)\Phi }_{22}^{23}(\vec{q})\\ -x\Phi_{21}^{31}(\vec{q}) & -x\Phi_{22}^{32}(\vec{q}) & \omega^{2}m_{X}-\Phi_{22}^{33} \end{array}\right|=0,$$

with the solution of Equation (7) leading to the vibrational mode frequencies.

Extension of the above one-dimensional approach to simulate the lattice dynamics of three-dimensional ideal zb random A_x_B_1-x_O ternary alloys using GF theory [69] in the framework of a RIM [68] is trivial. Here, we have assumed that the mixed A_x_B_1-x_O crystal lattice forms (a) ideal pseudo-binary alloys in the entire composition range x, (probably in contrast to the reality), (b) the cation sublattice 2, which is structurally close to the virtual crystal lattice where A and B atoms are randomly distributed having the concentration of B (1 − x) and of A as x, (c) the anion sublattice 1 with O atoms remaining undistorted, and (d) the characteristic nearest-neighbor atomic distances, which follow Vegard’s law. It is to be noted that in GF methodology, no additional IFCs are required for the descriptions of wave vector-dependent phonon dispersions $\omega_{j}\left(\vec{q}\right)$ of A_x_B_1-x_O alloys in arbitrary crystallographic directions. To simulate the dynamical characteristics of pseudo-binary alloys using RIM (cf. Section 4.3), we have deliberated IFCs of the binary zb ZnO and BeO materials in a three-body framework. While this approach has allowed the calculations of phonon spectra for the optical, acoustic, and disorder-induced modes, the disorder-related broadening of spectral lines has been neglected.

## 4. Numerical Simulations Results and Discussions

In materials, the significance of lattice dynamics for understanding the interactions among the atoms is well known to those working in solid-state physics, electronics, optoelectronics, quantum electronics, superconductivity, etc. Phonons in LDHs can act as a guide to research on dimensionally confined modes, leading to the vibrational effects to design nano- and/or microelectronic device structures [16]. Following standard practices and using optimized set of RIM parameters (cf. Table 2), we have computed the lattice dynamics and thermodynamic properties of the binary zb ZnO, BeO, and the ternary Be_x_Zn_1-x_O alloys. A generalized GF method is adopted (cf. Section 3.2) by carefully incorporating the necessary IFCs (Table 2) of binary materials to study phonon characteristics of ternary alloys. The theoretical results are analyzed by comparing/contrasting them with the existing experimental and ab initio calculations [72,73,74,75,76,77,78,79,80].

### 4.1. Vibrational Properties of XO Materials

To understand the lattice dynamical properties of zb XO materials, the best fit values of IFCs (cf. Table 2) are used in Equation (1) to calculate the phonon dispersions $\omega_{j}\left(\vec{q}\right)$. The results of the simulated one-phonon DOS $g\left(\omega \right)$ are achieved by incorporating the phonon values at a mesh of 64,000 $\vec{q}$ points in the BZ. Standard practices are employed here for calculating the one phonon DOS $g\left(\omega \right)$ by setting the sampling widths of phonons at Δω = ω_LO(Γ)_/100.

#### 4.1.1. Lattice Dynamics and Density of States for zb ZnO

The basic understanding of thermal as well as electrical properties in terms of the low- and high-field carrier transport requires a precise knowledge of the vibrational modes of the bulk crystals. In a zb ZnO material, we have simulated the RIM results of the phonon dispersions $\omega_{j}\left(\vec{q}\right)$ along high-symmetry directions (Γ→ X→ K→ Γ→ L→ X→ W→ L). Our theoretical results of the phonon frequencies at high critical points in the BZ are compared/contrasted (see Table 3) against the existing experimental and/or ab initio calculations [54,60,61,62].

**Figure 2:**
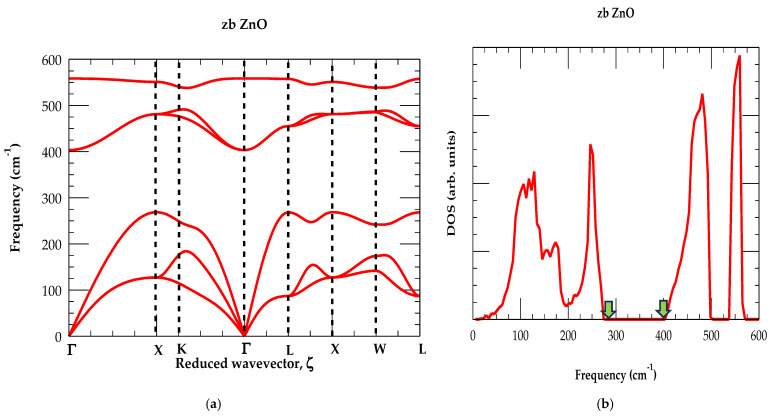
(**a**) Calculated rigid-ion-mode phonon dispersions using parameter values of Table 2 for the zb ZnO along high-symmetry directions. (**b**) Calculated rigid-ion-mode result of the one-phonon density of states for zb ZnO.

Close inspection of the optical and acoustic phonon dispersions (see Figure 2a) along the high-symmetry directions and DOS reported in Figure 2b has confirmed that they are affected by the atomic masses of the O (16.00 amu) and Zn (63.58 amu) atoms, respectively. Obviously, there are no negative frequencies in our study that confirm that the zb ZnO is dynamically stable. It corroborates the experimental claims of its synthesizability [16]. The study has provided non-degenerate longitudinal acoustic $\omega_{LA}$ and longitudinal optical $\omega_{LO}$ phonons, while the transverse acoustic $\omega_{TA}$ and transverse optical $\omega_{TO}$ modes are doubly degenerate. The RIM study has predicted the correct optical phonon splitting $\Delta \omega_{opt}$ (≡ $\omega_{LO(\Gamma )}$ − $\omega_{TO(\Gamma )}$) at the center of the BZ [54]. This phonon separation $\Delta \omega_{opt}$ is required for estimating the effective transverse charge $e_{T}^{*}$. Our systematic calculations have also offered a strong justification to the existing first-principle results [54,55,66] about the flatness of $\omega_{TA(X)}$ and $\omega_{TA(L)}$ branches as they fall below the region of the phonon band gap region.

In Figure 2b, we have displayed our simulated results of the one-phonon DOS $g\left(\omega \right)$ for the zb ZnO. A wide phonon gap (~275–405 cm^−1^) between the acoustic and optical branches is clearly identified. The phonon gap region is indicated by using the green-colored arrows. For zb ZnO, the RIM lattice dynamical calculations of phonon frequencies (all in cm^−1^) at the major critical points (Γ, X, L) in the BZ are summarized in Table 3. Comparison of our calculations are made with the existing INS and RSS experimental [54] data as well as the ab initio calculations [54,55,61,62]. Clearly, our phonon values have agreed reasonably well (see Table 3) with those of Serrano et al. [54,55]. Obviously, the results of the phonon dispersion curves $\omega_{j}(\vec{q})$ and DOS $g\left(\omega \right)$ for the zb ZnO reported by Chibueze [61] and Zafar et al. [62] are either ambiguous and/or questionable.

#### 4.1.2. Lattice Dynamics and Density of States for zb BeO

**Figure 3:**
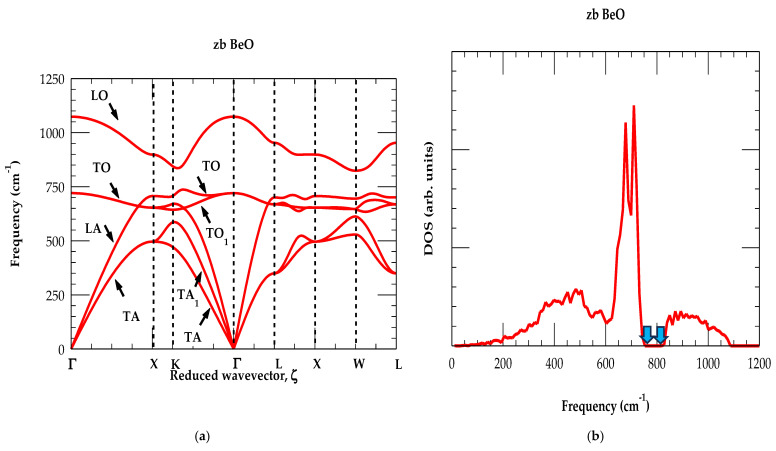
(**a**) Calculated rigid-ion-mode phonon dispersions using parameter values of Table 2 for the zb BeO along high-symmetry directions. (**b**) Calculated rigid-ion-mode result of the one-phonon density of states for zb BeO.

For zb BeO, the RIM results of $\omega_{j}\left(\vec{q}\right)$ are displayed in Figure 3a along the high-symmetry directions of the BZ. Calculations of one-phonon DOS $g\left(\omega \right)$ are also reported in Figure 3b. In the absence of experimental data, the phonon values (see Table 3) are compared at a few critical points against the existing ab initio data [60]. Contrasting the $\omega_{j}\left(\vec{q}\right)$ results of BeO with zb BN, diamond (C) materials [81,82,83] have revealed some interesting features. In zb BeO, the zone-center optical phonons has exhibited a very large $\Delta \omega_{opt}$ $\equiv {(\omega }_{LO\left(\Gamma \right)}-\omega_{TO\left(\Gamma \right)})$ splitting 353 cm^−1^. Moving away from the Γ-point (i.e., the center of BZ), the $\omega_{LO}\left(\omega_{TO}\right)$ modes offered high (low) dispersive behavior along the major high-symmetry [Γ→X], [X→Γ], [Γ→ L] directions. The calculated one-phonon DOS $g\left(\omega \right)$ of zb BeO has revealed a broad band between 825 cm^−1^–1075 cm^−1^ (see Figure 3b), showing a minimum phonon gap appearing in the region between 744 cm^−1^–825 cm^−1^ (see blue-colored arrows).

Unlike most other zb II–VI compounds, including zb ZnO, the flatness in $\omega_{TA}$ phonon branches near the X and L critical points are absent in the zb BeO. For large $\vec{q}$, the RIM simulation of $\omega_{j}\left(\vec{q}\right)$ has also revealed an atypical behavior of $\omega_{LA}$ phonon branches along the Γ→X and Γ→L directions. That is, the $\omega_{LA}$ modes exhibit higher frequencies than the $\omega_{TO}$ phonons (i.e., $\omega_{LA}>\omega_{TO}$ (see Figure 3a)). Accordingly, in $g\left(\omega \right),$ (see Figure 3b) no clear phonon gap appears between the acoustic and optical branches. Interestingly, identical trends are noticed in $\omega_{j}\left(\vec{q}\right)$ and $g\left(\omega \right)$ for the BN and C materials [81,82,83]. As compared to ZnO, the distinct phonon features in BeO can be ascribed to the changes in bond lengths for affecting the stiffness in the bonds as well as bond ionicity (or covalency). Our lattice vibrational results provided a strong justification to an earlier assertion that BeO could exhibit structural, elastic, and dynamic characteristics like those of the super hard BN and C materials [17]. The theoretical study of eigenvectors for the zb BeO material [60] is linked to the vibration of the lighter Be atom to $\omega_{LO(X)}$ and the heavier O oscillation to $\omega_{LA(X)}$ phonon.

### 4.2. Thermodynamic Properties of XO Materials

In XO materials, the Debye temperature is a valuable physical parameter for comprehending the excitations of lattice phonons [73] and appraising the interatomic forces. Knowledge of specific heat offers a strong measure for evaluating the storage of thermal energy in all the active atomic oscillations. These quantities have played vital roles in selecting MQWs and SLs for designing diverse nano- and micro-structured devices [16]. In semiconductors, the values of $\Theta_{D}\left(T\right)$ at low T are commonly obtained by exploiting the heat capacity $C_{v}\left(T\right)$ measurements via the speed-of-sound or heat flux calorimetry [73]. Low-temperature heat capacity $C_{v}\left(T\right)$ experiments in XOs (X = Be and Zn) are essential for understanding the phase transformation and construction of phase diagrams in the low-temperature regions of equilibrium P and T. However, experimental constraints have remained for accurately probing $C_{v}\left(T\right)\;at\;low\;T\;and\;high\;P$ [73].

Thus, very few measurements exist on $\Theta_{D}\left(T\right)$ and $C_{v}\left(T\right)$ for the zb ZnO and BeO materials [16]. Theoretically, the calculated phonon dispersions and one-phonon DOS g(ω) are used here to assess the thermodynamic traits [e.g., Debye temperature, $\Theta_{D}\left(T\right)$, heat capacity $C_{v}\left(T\right)$] of XOs for comprehending their optical characteristics [16]. Thermodynamic and optical features are frequently used to assess how these materials respond to the temperature change. These characteristics have also helped engineers to design various devices for thermoelectric, energy storage, and high-temperature application needs [16].

#### 4.2.1. Debye Temperature of Binary zb ZnO and BeO

Although several research groups have employed different theoretical methods to simulate the structural, P-, and/or T-dependent lattice-, elastic-, and thermodynamic characteristics—the reported results on $\Theta_{D}\left(T\right)$ have exhibited sizable discrepancies [72,73,74,75,76,77,78,79,80]. By incorporating RIM phonon dispersions $\omega_{j}\left(\vec{q}\right)$ and $g\left(\omega \right)$ of XO materials and using Equations (4) and (5), we have systematically calculated $C_{v}\left(T\right)$ to assess $\Theta_{D}\left(T\right)$.

**Figure 4:**
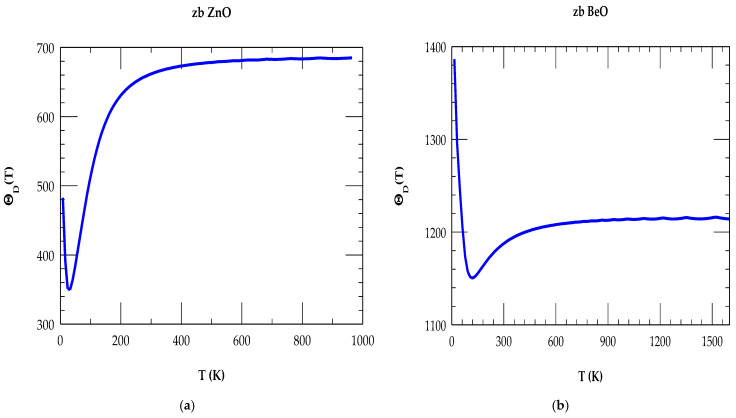
(**a**) Calculated rigid-ion-model results of Debye temperature $\Theta_{D}$(T) vs. T for zb ZnO using IFCs values of Table 2. (**b**) Same key as of Figure 3a but for zb BeO.

The RIM simulations of T-dependent $\Theta_{D}\left(T\right)$ for zb ZnO and BeO materials are displayed in Figure 4a,b for 0 ≤ T ≤ 1600 K, respectively. In a wide range of T, our RIM calculations of $\Theta_{D}$(T) for zb ZnO and zb BeO materials have revealed identical trends. At low T, the calculations of $\Theta_{D}\left(T\right)$ have agreed reasonably well with the general behavior. For instance, in zb BeO (zb ZnO) materials of lower (higher) density and higher (lower) melting point, $\Theta_{D}\left(T\to 0\right)$ exhibits a large ~1400 K (small ~483 K) value. The decrease in theoretical values of $\Theta_{D}$(T→0) (cf. Figure 4a,b) from the zb BeO to ZnO is linked to the increase in X (cation) atomic mass [i.e., from Be (9.01 amu) → Zn (63.58 amu)]. The higher $\Theta_{D}\left(T\right)$ of zb BeO indicates an exceptional thermal stability and its resistance to thermal expansion [74,75].

For nanostructured ZnO and BeO materials, the $\Theta_{D}\left(T\right)$ value is projected to be lower than the reported values of their bulk counterparts [16]. It is simply due to an increased surface area and enhanced thermal vibrations at the surface as compared to the bulk interior. The size-dependent Debye temperature is a consequence of the increased surface-to-volume ratio in the nanomaterials, leading to the altered thermal and elastic properties [72,73,74,75,76,77,78,79,80].

In Table 4, we have compared our RIM results of thermodynamic quantities $(\Theta_{D}\left(T\right)$ and $C_{v}\left(T\right))$ at selected temperatures for the zb ZnO and BeO materials with the existing experimental/theoretical data [16,77,78,79,80]. Large discrepancies with significantly lower values of Debye temperatures in zb ZnO reported in [16] are possibly related to the diverse analytical expressions used by different authors. In Section 4.2.2, we have compared and contrasted our T-dependent RIM results of $C_{v}\left(T\right)$ for the zb XO binary materials with the existing experimental and theoretical [72,73,74,75,76,77,78,79,80] data.

#### 4.2.2. Specific Heat of Binary zb ZnO and BeO

Earlier, in a physical property measurement system and using a calibrated heater-thermometer platform, the specific heat $C_{v}\left(T\right)$ measurements were performed on bulk rs-ZnO, w-ZnO, and BeO materials between 310 K > T > 2 K [77,78]. In Table 4, a comparison of the thermodynamic quantities is made using RIM for the zb ZnO and BeO with existing experimental and ab initio calculations [16,77,78,79,80].

**Figure 5:**
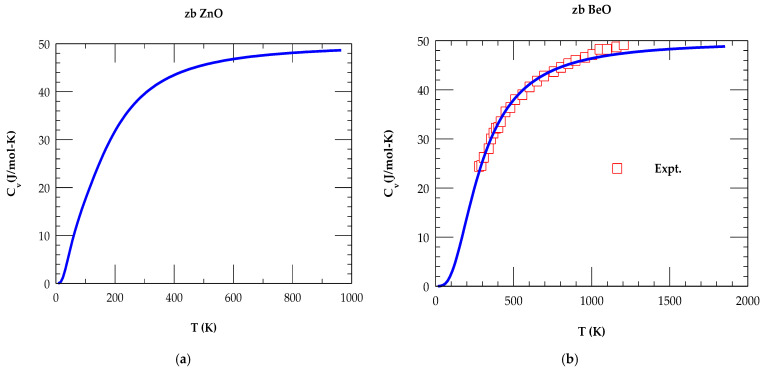
(**a**) Calculated rigid-ion mode results of specific heat $C_{v}\left(T\right)\;vs.$ T for zb ZnO using IFCs values of Table 2. (**b**) Same key as of Figure 4a, but for zb BeO, the red-colored open squares represent the experimental data [77,78].

The results of our T-dependent simulations of $C_{v}\left(T\right)$ are displayed in Figure 5a,b, respectively, for zb ZnO and BeO. In each material, the shape of the calculated $C_{v}\left(T\right)$ is seen changing appropriately with the increase in T. At higher T, $C_{v}\left(T\right)$ approaches the Dulong–Petit limits (~50 J/mol–K). For a given T, the $C_{v}\left(T\right)$ $\left(\Theta_{D}\left(T\right)\right)$ in BeO (ZnO) decreased (increased) with a decrease in anion mass, in very good agreement with the ab initio calculations [78]. Except for the large differences in the values reported by Kamenev et al. [76], our calculations of $\Theta_{D}$ (T → 0 ~ 483 K), $\Theta_{D}$ (T → 297 ~ 661 K), and $\Theta_{D}$ (T→ ∞ ~ 686 K) for the zb ZnO and BeO of $\Theta_{D}$ (T → 0 ~ 1390 K), $\Theta_{D}$ (T → 297 ~ 1187 K) and $\Theta_{D}$ (T→ ∞ ~ 1214 K) agreed reasonably well with the experimental [77,78] and theoretical [16] data.

In Section 4.3.1 and Section 4.3.2, we have reported our RIM results in the GF framework on the composition-dependent $\omega_{j}\left(\vec{q}\right),\Theta_{D}\left(T\right)$ and $C_{v}\left(T\right)$ for the ternary Be_x_Zn_1-x_O alloys.

### 4.3. Phonons in Ternary Be_x_Zn_1-x_O Alloys

Controlling heat transport at the film interfaces in LDHs-based ZnO/(Be)ZnO structures is critical for achieving the energy conversions required in the growing needs of energy management applications for developing thermoelectric, nano- and microelectronic, photonic, and plasmonic devices [16]. In the layered structures, the mechanisms of interfacial heat transport can be easily manipulated by adjusting the phonon modes and their scattering. Understanding the contributions of heat transport from each layer is important to engineering novel micro- and nano-electronic/photonic devices for achieving their optimal thermal performance.

In this context, the lattice vibrations of Be_x_Zn_1-x_O ternary alloys and BeO/ZnO SLs have played crucial roles for manipulating high performance in the flexible UV photodetectors for different electronic devices in thermal management applications. 

#### 4.3.1. Lattice Dynamics and Density of States of Be_x_Zn_1-x_O

In an earlier study [18], we have considered a modified random-element iso-displacement (MREI) model for comprehending the composition-dependent long-wavelength optical phonons ($\vec{q}$ → 0) in the zb Be_x_Zn_1-x_O ternary alloys. The calculation [18] has predicted a two-phonon mode behavior in excellent agreement with recent results reported for wz Be_x_Zn_1-x_O ternary alloys [59].

**Figure 6:**
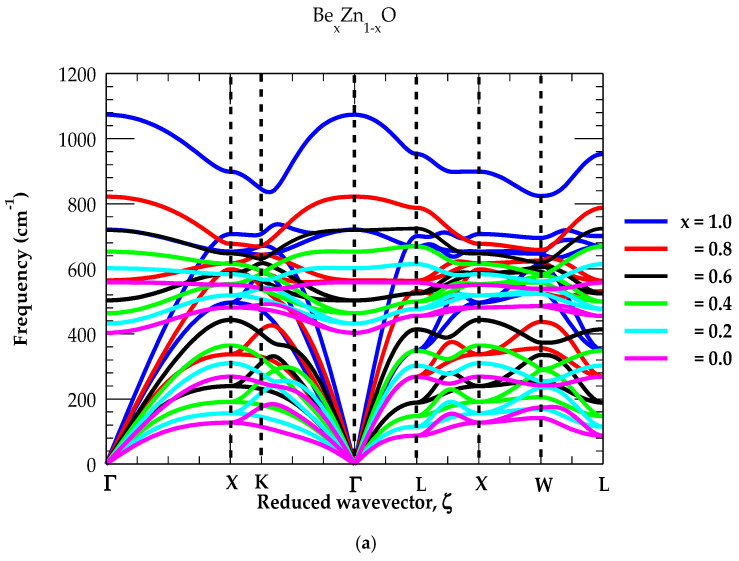

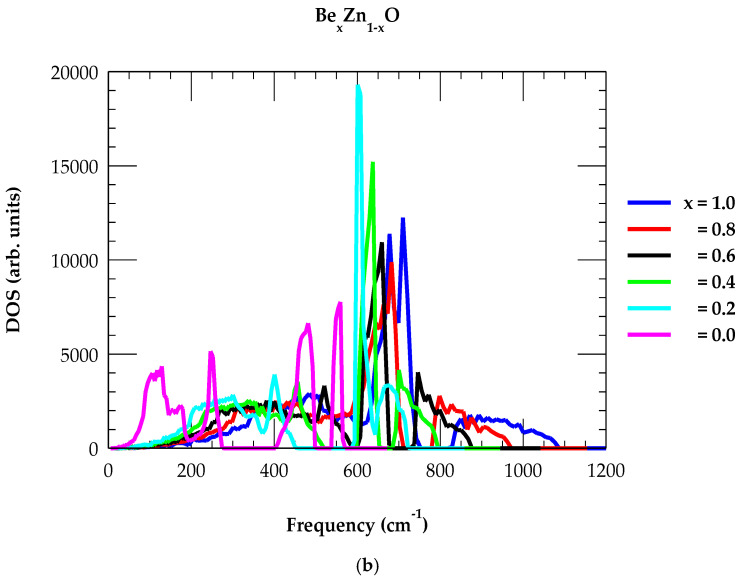
(**a**) Calculated rigid-ion-model results of phonon dispersions of ternary Be_x_Zn_1-x_O alloys. IFCs values of Table 2 were used and the methodology outlined in Section 3.2. was adopted. (**b**) Same key as of Figure (**a**) but for the density of states of ternary Be_x_Zn_1-x_O alloys.

By appropriately including the IFCs (see Table 2) of bulk binary zb ZnO and BeO materials in a methodical GF [69] approach (cf. Section 3.2), we report here our simulated x-dependent (≡ 0, 0.2, 0.4, 0.6, 0.8 and 1) results of phonon dispersions $\omega_{j}\left(\vec{q}\right)$ and one-phonon DOS $g\left(\omega \right)$ for Be_x_Zn_1-x_O ternary alloys (see Figure 6a,b).

Due to the significant difference in the phonon frequencies of BeO and ZnO materials, the results for Be_x_Zn_1-x_O alloys have revealed ZnO-like optical modes hybridizing with the BeO-like LA modes. We strongly feel that these features will enhance phonon–phonon scattering and lower the thermal conductivity of Be_x_Zn_1-x_O ternary alloys [16]. From Figure 6a,b, one may also note that, in the limiting situations (i.e., x → 0 and x → 1), the RIM phonon features of the ternary Be_x_Zn_1-x_O alloys transform to those of the binary zb ZnO and BeO materials.

#### 4.3.2. Debye Temperature and Specific Heat of Be_x_Zn_1-x_O

**Figure 7:**
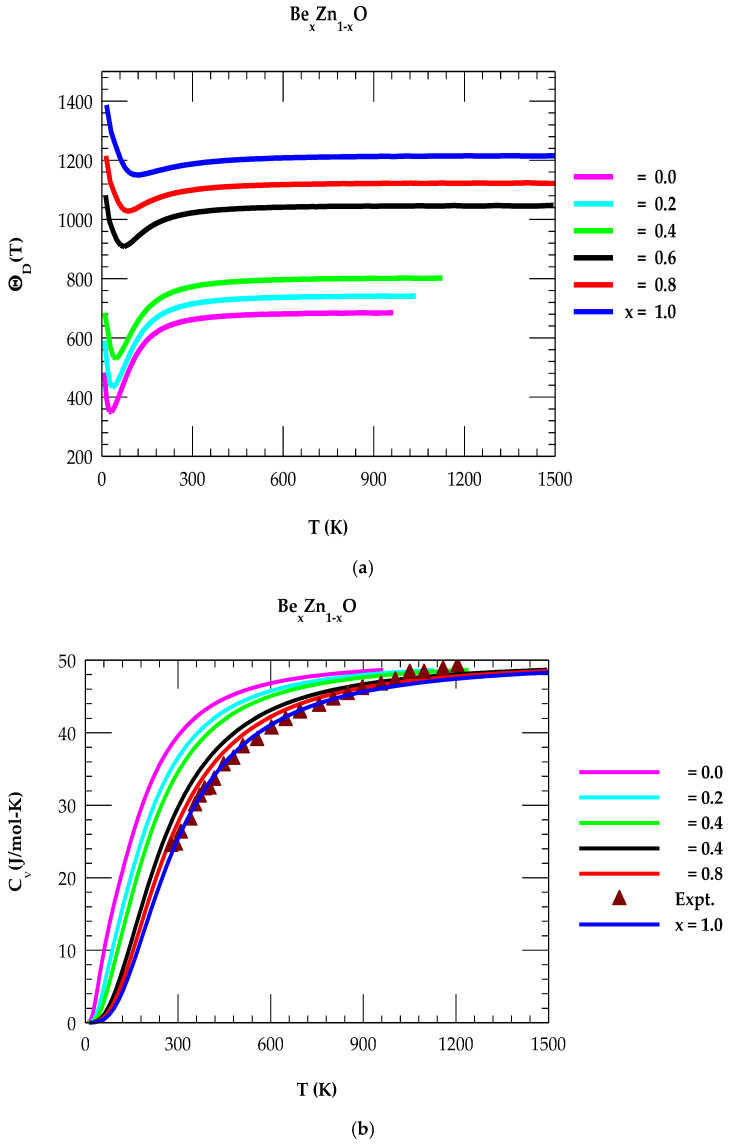
(**a**) Calculated rigid-ion model results of Debye temperature $\Theta_{D}$(T) vs. T for zb Be_x_Zn_1-x_O using IFCs values of Table 2 and methodology described in Section 3.1.4 and Section 3.2. (**b**) Calculated results of specific heat $C_{v}$(T) vs. T for zb Be_x_Zn_1-x_O using the IFCs values of Table 2 and the methodology described in Section 3.1.4 and Section 3.2. The result for x = 1 is compared with the experimental data.

By using the composition-dependent phonon dispersions $\omega_{j}\left(\vec{q}\right)$ and DOS $g\left(\omega \right)$ in Equations (4) and (5), we have reported our simulated results of $\Theta_{D}$(T) and $C_{v}\left(T\right)$ for Be_x_Zn_1-x_O ternary alloys (see Figure 7a,b).

The Debye temperature of a solid signifies its characteristic temperature that relates to the speed of sound and density of the material. It represents the maximum energy that phonons (lattice vibrations) can have at a given temperature. The lighter Be atom in BeO leads to higher vibrational frequency at lower temperatures as compared to lower vibrational frequency for the heavier Zn atom in ZnO. Consequently, for zb ZnO, it causes a lower Debye temperature. Obviously, the strength of chemical bonds between Be-O and Zn-O impacts their vibrational mode frequencies and thus $\Theta_{D}$(T). Stronger bonds typically lead to higher vibrational frequencies and higher $\Theta_{D}$(T). Again, the ratio of Be to Zn in Be_x_Zn_1-x_O alloys are seen affecting $\Theta_{D}$(T). While $\Theta_{D}$(T) is considered a property of materials, it is alsodependent on T, especially at higher T, due to thermal expansion and changes in lattice parameters. The x-dependent results of $\Theta_{D}$(T) for the Be_x_Zn_1-x_O reported in Figure 7a have clearly revealed a higher value for BeO (x → 1) than ZnO (x → 0).

In the Debye model, the heat capacity $C_{v}\left(T\right)$ results are reported in Figure 7b for the Be_x_Zn_1-x_O ternary alloys. Obviously, the $C_{v}\left(T\right)$ of Be_x_Zn_1-x_O is influenced by several factors, including the alloy composition, x, T, and the electronic and phonon structure [16]. The perusal of Figure 7b has clearly shown that $C_{v}\left(T\right)$ starts at a zero value; it increases with T^3^ at low T for all x and gradually attains the Dulong–Petit limit (~ 50 J/mol–K) at higher T. Again, from Figure 7b, our results have perceived that in the temperature range (300 K < T < 1000 K), the $C_{v}\left(T\right)$ depends both on T and x in Be_x_Zn_1-x_O—exhibiting lower values for ZnO than BeO. The outcome of our study has not only concurred a satisfactory behavior comparable with the experimental data for XO [54,60] materials but also corroborated the first-principle calculations [60] known for several binary and ternary alloy semiconductors. Obviously, the specific heat results of Be_x_Zn_1-x_O can influence their suitability in various applications, namely thermal insulation, heat sinks, and energy storage systems. 

## 5. Summary and Concluding Remarks

Understanding the electronic, structural, and thermal properties of novel ZnO and BeO materials requires a precise knowledge of their vibrational modes in terms of low- and high-field carrier transport [16]. Lattice dynamics and thermal properties of materials are known to play the crucial roles in solid state physics, materials science, and electronics. The valuable material characteristics include $\omega_{j}\left(\vec{q}\right)$, thermal conductivity κ(T), heat capacity $C_{v}\left(T\right)$, Debye temperature $\Theta_{D}$(T), thermal expansion α(T), etc. These traits are crucial for creating novel materials and designing efficient device systems that can handle temperature changes and heat transfer. BeO is a WBG semiconductor with high thermal conductivity, while ZnO has many unique electrical properties suitable for developing optoelectronics, power electronics, and thermal management systems. Complete phonon dispersions $\omega_{j}\left(\vec{q}\right)$ of bulk zb ZnO are available by INS spectroscopy [54,55]. Many attempts are also made to obtain the vibrational modes of ultrathin ZnO epifilms. Phonon frequencies of ZnO epifilms derived by RSS and FTIR spectroscopies are consistent with those of the bulk ZnO using INS [54,55].

Unfortunately, similar measurements of $\omega_{j}\left(\vec{q}\right)$ for both the bulk zb BeO and/or thin epifilms are lacking. Absolutely no efforts have been made to achieve $\omega_{j}\left(\vec{q}\right)$ for the ternary Be_x_Zn_1-x_O alloys. A deeper understanding of phonon dispersions is necessary for binary ZnO, BeO, and ternary Be_x_Zn_1-x_O materials by using realistic lattice dynamical models. The information on lattice dynamics and thermal properties can be crucial for developing LDHs-based advanced flexible micro- and nano-optoelectronics [16]. By adopting realistic RIM, we have reported the results of our methodical study to predict the structural, phonon, and thermodynamical properties of novel zb ZnO and BeO. The IFCs for these materials are systematically included in the GF [69] methodology for simulating $\omega_{j}\left(\vec{q}\right),\;g\left(\omega \right),\;\Theta_{D}$(T), and $C_{v}\left(T\right)$ for zb Be_x_Zn_1-x_O ternary alloys. The results of our RIM calculation are compared and contrasted reasonably well with the limited experimental and ab initio calculations. Due to the significant variation in the bond strength of Be-O from Zn-O, the phonon frequencies of BeO and ZnO materials are seen quite different. The calculated $\omega_{j}\left(\vec{q}\right)$ for the Be_x_Zn_1-x_O ternary alloys have revealed ZnO-like optical modes hybridizing with the BeO-like LA modes. In BeZnO, the intermixing of such phonons represents a fundamental interaction between the vibrational characteristics of two constituent materials. This feature can certainly affect the overall properties of LDHs-based structures, which, in turn, can impact the performance of optoelectronic devices due to thermal transport and electron–phonon interactions.

Heat dissipation in micro and nanoelectronic devices has been and still is a major concern among scientists and engineers. In MQW- and SL-based structures involving the ternary Be_1-x_Zn_x_O alloy, epifilms have predicted a reduction in the thermal conductivity to affect heat dissipation and stability of various electronic devices [16]. The introduction of Zn atoms into BeO lattice creates a disordered structure that enhances phonon scattering and disrupts heat transport. Consequently, in Be_1-x_Zn_x_O alloys, one would expect a reduction in thermal conductivity [16]. Low thermal conductivity of ultrathin alloy Be_1-x_Zn_x_O films can be used to reduce heat transfer and prevent overheating of various micro- and nanoelectronic device modules. Other applications of the ternary Be_1-x_Zn_x_O alloy epifilms include creating thermal barriers, insulating electronic components, and developing the solar-blind detectors. Recently, Be_x_Zn_1-x_O alloys have been used for creating insulation layers to prevent heat conduction from different heat-generating components, including transistors and/or microprocessors. As a material, Be_1-x_Zn_x_O can be used in solar-blind UV detectors, as they are sensitive to UV light and insensitive to visible light. Certainly, it will improve the stability and reliability of UV detectors under high-temperature operations.

In summary, our methodical results of RIM calculations on the lattice dynamics and thermal properties of Be_1-x_Zn_x_O alloys are important. We strongly feel that the theoretical predictions made here of the phonon and thermodynamic characteristics for the novel binary zb ZnO, zb BeO, and Be_1-x_Zn_x_O ternary alloys will encourage experimentalists to perform similar measurements to check our theoretical conjectures. In MQW- and SL-based structures involving BeZnO epifilms, one expects a reduction in the thermal conductivity, which can affect the heat dissipation and reliability of various electronic devices [16]. The low thermal conductivity of Be_1-x_Zn_x_O-based structures is a valuable property that should be exploited in a variety of applications, including thermal barriers, electronic insulation, and advanced UV detectors. 

## Figures and Tables

**Table 1:** Critical-point phonon frequencies (cm^−1^) of zb ZnO (column 3) and BeO (column 5) and lattice constants $a_{o}$ in (Å) elastic constants $c_{ij}$ 10^11^dyn/cm^2^. These parameters [54,60] are used for optimizing the rigid-ion model (RIM) interatomic force constants (see Table 2) for the binary materials.

Parameters	zb ZnO (Our) ^(a)^	Others ^(b)^	zb BeO (Our) ^(a)^	Others ^(c)^
$\omega_{LO(\Gamma )}$	558	525	1074	1060
$\omega_{TO(\Gamma )}$	403	403	721	683
$\omega_{LO(X)}$	551	555	899	900
$\omega_{TO(X)}$	487	444	653	655
$\omega_{LA(X)}$	269	268	707	708
$\omega_{TA(X)}$	128	80	496	493
$\omega_{LO(L)}$	561		953	902
$\omega_{TO(L)}$	443		669	663
$\omega_{LA(L)}$	264		701	702
$\omega_{TA(L)}$	93		349	310
$a_{o}$	4.504	4.520–4.666	3.80	3.72–3.83
$c_{11}$	19.19	15.1–19.3	34.2	34.2
$c_{12}$	14.79	11.06–15.8	13.9	14.8
$c_{44}$	7.34	7.4–12.8	21.7	20.8

^(a)^ Our; ^(b)^ Ref. [54]; ^(c)^ Ref. [60].

**Table 2:** In the notations of Ref. [68] and following Ref. [71], we have obtained the optimized sets of rigid-ion-model (RIM) parameters [A, B, C_1_, C_2_, D_1_, D_2_, E_1_, E_2_, F_1_, F_2_ (10^5^ dyn/cm)] at ambient pressure for novel zb XO (X = Zn and Be) materials. The term Z_eff_ is the effective charge (see text).

RIM ^(a)^ Parameters	zb ZnO ^(b)^	zb BeO ^(b)^
A	−0.40207	−0.62022
B	−0.395	−0.55000
C_1_	−0.0540	−0.06650
C_2_	−0.0490	−0.09300
D_1_	−0.0088	−0.04144
D_2_	−0.0900	−0.14900
E_1_	−0.0300	−0.10000
E_2_	0.0600	0.04000
F_1_	−0.0360	0.15500
F_2_	0.12300	−0.12500
Z_eff_	0.9435	1.0133

^(a)^ Ref. [68]; ^(b)^ Ref. [71].

**Table 3:** Comparison of our RIM-calculated phonon frequencies (cm^−1^) of zb ZnO and BeO at critical point in the BZ with the existing experimental [54,55] and first-principle calculations [54,60,61,62].

Modes	zb ZnO (Our) ^(a)^	Others ^(b), (c)^	zb BeO (Our) ^(a)^	Others ^(d)^
$\omega_{LO(\Gamma )}$	558	525, 517	1074	1060
$\omega_{TO(\Gamma )}$	403	403, 367	721	683
$\omega_{LO(X)}$	551	555, 495	899	900
$\omega_{TO(X)}$	487	444, 442	653	655
$\omega_{LA(X)}$	269	268, 270	707	708
$\omega_{TA(X)}$	128	80, 121	496	493
$\omega_{LO(L)}$	561		953	902
$\omega_{TO(L)}$	443		669	663
$\omega_{LA(L)}$	264		701	702
$\omega_{TA(L)}$	93		349	310

^(a)^ Ours; ^(b), (c)^ Refs. [54,55,61,62]; ^(d)^ Ref. [60].

**Table 4:** Comparison of the thermodynamical ($\Theta_{D}$, $C_{v}$) properties of zb ZnO and BeO with the existing experimental and first-principle calculations. The Debye temperature is in K while $C_{v}$ is in the units of (J/mol-K).

	zb ZnO (Our) ^(a)^	Others ^(b), (c)^	zb BeO (Our) ^(a)^	Others ^(d)^
$\Theta_{D}\left(0\right)$	483	418, 519	1390	1270; 1280
$\Theta_{D}\left(297\right)$	661		1187	1188
$\Theta_{D}^{min}(T)$	350 @ 32 K		1150 @ 124 K	
$\Theta_{D}\left(HighT\right)$	686 @ 970 K		1214 @ 1000 K	
$C_{v}\left(100\right)$	16.97	15.87	3.17	
$C_{v}\left(297\right)$	39.43	37.5	24.78	25.51–26.11
$C_{v}\left(HighT\right)$	48.65 @ 960 K	48.7 @ 960	48.83 @ 1850 K	48.72 @ 1150 K

^(a)^ Our; ^(b), (c)^ Refs. [16,79,80]; ^(d)^ Refs. [77,78].

## Data Availability

The data that support the findings of this study are available from the corresponding author upon reasonable request.
